# The Triggering Receptor Expressed on Myeloid Cells 2: “TREM-ming” the Inflammatory Component Associated with Alzheimer's Disease

**DOI:** 10.1155/2013/860959

**Published:** 2013-03-06

**Authors:** Troy T. Rohn

**Affiliations:** Department of Biological Sciences, Boise State University, Science Building, Room 228, Boise, ID 83725, USA

## Abstract

Alzheimer's disease (AD) is an age-related neurodegenerative disorder characterized by a progressive loss of memory and cognitive skills. Although much attention has been devoted concerning the contribution of the microscopic lesions, senile plaques, and neurofibrillary tangles to the disease process, inflammation has long been suspected to play a major role in the etiology of AD. Recently, a novel variant in the gene encoding the triggering receptor expressed on myeloid cells 2 (TREM2) has been identified that has refocused the spotlight back onto inflammation as a major contributing factor in AD. Variants in TREM2 triple one's risk of developing late-onset AD. TREM2 is expressed on microglial cells, the resident macrophages in the CNS, and functions to stimulate phagocytosis on one hand and to suppress cytokine production and inflammation on the other hand. The purpose of this paper is to discuss these recent developments including the potential role that TREM2 normally plays and how loss of function may contribute to AD pathogenesis by enhancing oxidative stress and inflammation within the CNS. In this context, an overview of the pathways linking beta-amyloid, neurofibrillary tangles (NFTs), oxidative stress, and inflammation will be discussed.

## 1. Defining Alzheimer's Disease

Alzheimer's disease (AD) is classified as a neurodegenerative disorder affecting neurons of the brain that are responsible for memory and higher cognitive functions. The brain consists of over a 100-billion neurons that specialize in the ability to transmit information to other cells, and thus constitute the basic working unit of the brain. Because cortical neurons, in general, do not have the capacity to regenerate, once neurons are lost and symptoms manifest, the process is essentially irreversible. In this manner, Alzheimer's is classified as a progressive neurodegenerative disease that can take anywhere from 5–20 years to run its course. The loss of these neurons is significant with affected individuals losing up to 50% mass of the brain over the course of the disease. The loss of these neurons leads to the symptoms of the disease including memory impairments, difficulties with language, inability to execute motor activities, and the overall decline in cognitive skills [1]. Dementia is the umbrella term describing the symptoms of AD, and AD is by far the leading cause of dementia in the United States, being responsible for over 70% of all known cases of dementia [2]. AD is a multifactorial disorder, whose causes remain largely unknown. Despite extensive research on genetic factors, the vast majority of Alzheimer's cases (>90%) are not directly linked to them [3]. Aging is the most well-established risk factor for the development of sporadic AD with incidence rates showing an exponential growth between the ages of 65 and 85 years, doubling every 5 years [3].

The national numbers on AD are alarming: currently one in eight older Americans has AD making it the sixth leading cause of death in the United States. An estimated 5.4 million Americans have AD, a figure that includes 5.2 million people age 65 and older [1]. Of those with AD, an estimated 4 percent are under the age 65, 6 percent are 64 to 74, 44 percent are 75 to 84, and 46 percent are 85 or older [1]. Of all of the major causes of death in the United States, including stroke, cancer, and heart disease, only Alzheimer's disease has shown a significant increase in mortality during the same time frame (2000–2008).

## 2. Pathology Associated with AD

AD is diagnosed based upon the extent of senile plaques composed of beta-amyloid and neurofibrillary tangles (NFTs) containing abnormally phosphorylated and truncated tau [4]. The preponderance of research to date suggest a pivotal role for beta-amyloid in the progression of AD, and collectively this concept has coined the beta-amyloid hypothesis [5]. In essence, this hypothesis stipulates that much of the pathology associated with AD is driven by an increased load of beta-amyloid in the brain of AD patients that can occur years before the first symptoms of the disease manifest. 

**Figure 1:**
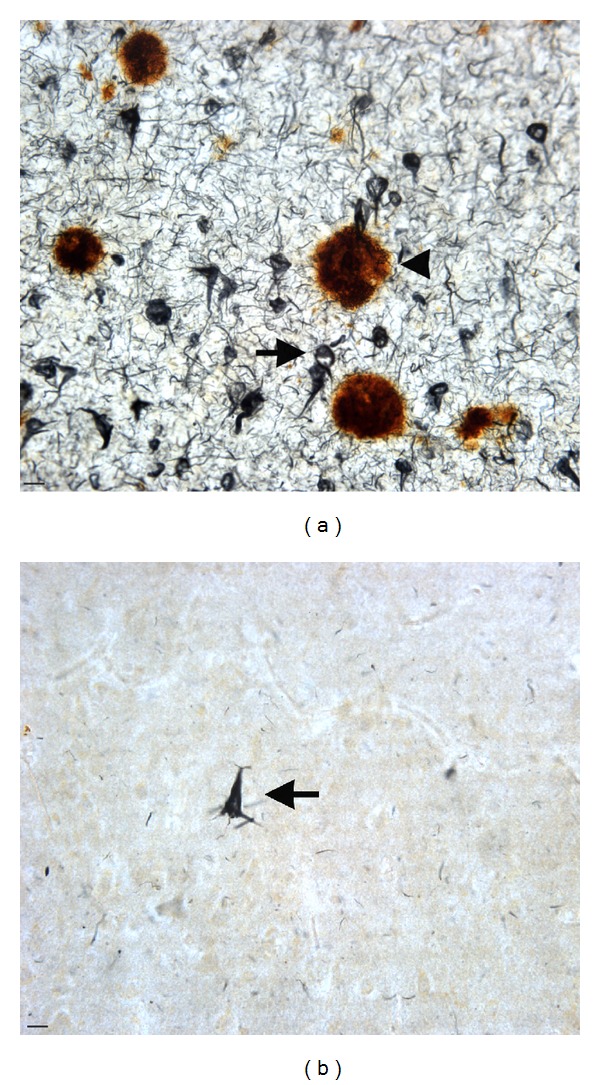
The microscopic trouble makers in Alzheimer's disease: senile plaques and neurofibrillary tangles. (a) In AD, widespread accumulation of extracellular beta-amyloid plaques is evident (arrowhead), together with the presence of an abundance of NFTs along with neuropil threads, which are composed principally of modified and aggregated tau (arrow). (b) For comparison purposes, an age-matched control brain is depicted indicating a complete absence of plaques. However, it is not uncommon to find an occasional tangle (arrow) in the normally aged brain although the numbers of tangles is minimal by comparison. Brain sections are representative staining of the hippocampus using an anti-beta-amyloid antibody to label plaques (brown) and anti-PHF-1 (black) to label NFTs. Scale bars are 10 *μ*m.

Beta-amyloid is formed following sequential cleavage of the amyloid precursor protein (APP) by two proteases, *β*-secretase and *γ*-secretase. Once formed, beta-amyloid has the propensity to self-aggregate into *β*-sheet structures that deposit extracellularly forming senile plaques (Figure 1). More recently, the beta-amyloid hypothesis has been modified to the “toxic beta-amyloid oligomer” hypothesis to reconcile the apparent lack of correlation between beta-amyloid in plaques and cognitive impairment [6]. This reformulation of the amyloid cascade hypothesis focuses on oligomeric aggregates of beta-amyloid as the prime toxic species causing AD in part because this form of beta-amyloid strongly correlates with the severity of dementia [7, 8]. In addition, this oligomeric form of beta-amyloid is highly toxic and is the trigger for the loss of synapses and neuronal damage [9, 10]. Given the strong support for the amyloid cascade hypothesis, many of the current therapeutic strategies now in clinical trials involve some aspect of modifying beta-amyloid production or clearance [11]. However, despite the overwhelming evidence supporting a role for beta-amyloid in AD, this hypothesis is currently under critical assessment due to the recent clinical trial failures based on the strategy of lowering the beta-amyloid levels in the AD brain [12]. For example, one strategy currently being investigated involves inhibiting gamma-secretase to limit the production of the beta-amyloid peptide derived from APP. One such compound, semagacestat, showed promise in early clinical trials, but a recent phase III trial involving over 2,600 participants was discontinued after failure to demonstrate efficacy. Compared to placebo, patients receiving semagacestat actually did worse both in daily function and cognition and were at higher risk of developing skin cancer [13].

 The other major pathological finding in AD is the presence of neurofibrillary tangles (NFTs) (Figure 1) [14]. NFTs are primarily composed of aggregated phosphorylated tau protein and are a clinical feature not just in AD but other diseases that are collectively referred to as “tauopathies” [15]. Tau normally functions to help maintain the stability of the cytoskeleton of neurons by binding to microtubules. However, upon hyperphosphorylation and posttranslational cleavage, tau loses its binding affinity for microtubules, leading to a destabilization of the cytoskeleton and self-assembly of tau into tangles of paired helical filaments (PHFs) [16]. Although not universally accepted, it has been proposed that NFTs may not be a central mediator of disease pathogenesis, but instead, NFTs may actually serve a protective rather than harmful function by providing a compensatory response mounted by neurons against oxidative stress [17].

## 3. Mechanisms of Neurodegeneration in AD

**Figure 2:**
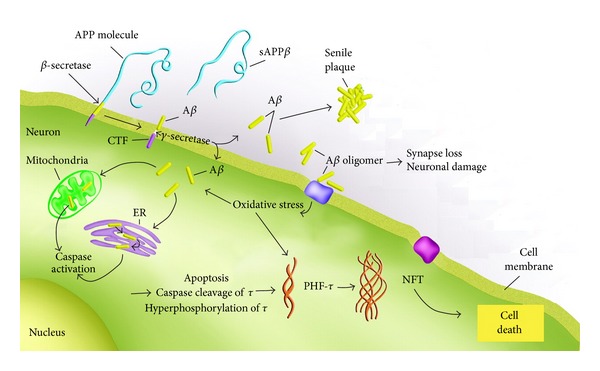
Putative pathway for neurodegeneration in Alzheimer's disease. According to the beta-amyloid hypothesis, the production of beta-amyloid represents the first step in the entire process following the sequential cleavage of APP by *β*-secretase and *γ*-secretase. Beta-amyloid in turn may lead to NFT formation and eventual cell death by promoting oxidative stress and caspase activation through initiation of the mitochondrial-mediated pathway of apoptosis. The activation of caspases results in cleavage of critical cellular proteins including tau, leading to its modification and hyperphosphorylation, a key prerequisite for filament formation. See main text for details.

 According to the beta-amyloid hypothesis, the accumulation and aggregation of beta-amyloid into toxic soluble oligomers is the first step leading to neuronal degeneration in AD [18]. Specifically, an important early molecular step is the lost of synapses, which correlates highly with the initial memory impairment observed in AD [19, 20]. Intensive research over the last two decades has examined potential pathways activated by beta-amyloid aggregates that lead to synaptic dysfunction, NFT formation, and eventual cell death.  Figure 2 summarizes some of the major findings on beta-amyloid-induced toxicity that begin with either beta-amyloid activation of apoptotic pathways or promotion of oxidative stress. With regards to apoptosis, since the early nineties, studies have supported a general role for apoptosis in AD [21–23]. In addition, the activation of caspases, including caspase 3, 6, 8, and 9, has been documented in the AD brain [24–30]. In turn, evidence suggests that once activated, caspases may cleave critical cellular proteins in AD including APP, actin, fodrin, glial acidic fibrillary protein, beclin-1, and tau [28, 31–35]. Importantly, several studies have suggested that caspase activation and cleavage of tau may precede and contribute to the formation of NFTs [31, 32, 36]. 

 An interlinking step between beta-amyloid and NFTs could be the promotion of oxidative stress. Oxidative stress either through lipid peroxidation or mitochondrial disruption is an early feature found in AD [37–39]. In addition, oxidative stress may contribute to the activation of apoptosis through both the extrinsic and intrinsic pathway [40, 41]. Finally, tau phosphorylation is upregulated by oxidative stress [42] and tau filaments are modified by products of oxidative stress [43–45]. Oxidative stress also activates several kinases that have been implicated in the hyperphosphorylation of tau including glycogen synthase kinase-3 (GSK3), Jun-N-terminal kinase (JNK), and mitogen-activated protein kinases (MAPKs) [46, 47]. 

## 4. Inflammation in AD

Brain inflammation is a pathological hallmark of AD [48, 49]. In this regard, numerous studies have supported a definitive role for inflammation in AD, with a key feature being the presence of activated microglia [50–52] and reactive astrocytes found within senile plaques [53–55]. Epidemiological studies have also pointed to inflammation as central to AD, indicating that the long-term use of anti-inflammatory drugs is linked with reduced risk of developing the disease [56]. Microglia are key players in mediating immune responses in the CNS functioning as the resident macrophages of the CNS and as such contribute to a healthy CNS by attacking and removing potential pathogens and cell debris and by secreting tissue rebuilding factors [57]. 

 Interestingly, the link between the activation of microglia and inflammation may be beta-amyloid. Thus, beta-amyloid is a potent inducer of microglia activation [52, 58–60], and one important role of microglia is to clear beta-amyloid deposits out of the AD brain (for recent review, see [61]). 

One potential caveat with inflammation is determining cause and effect. Does AD cause inflammation? does the dysregulation of immune system pathways trigger the disease process? Alternatively, although chronic inflammation may be a driving force in disease pathogenesis, it also may serve as a beneficial response at least early on during the course of AD. Finally, it is possible that inflammation could simply be a byproduct of the disease process and may not effectively alter its course. A recent discovery has now addressed this issue and has unequivocally put inflammation in general and specifically the microglial response center stage.

## 5. Triggering Receptor Expressed on Myeloid Cells 2 (TREM2)

 TREM2 is expressed on the cell membrane of many types of immune cells including macrophages, dendritic cells, osteoclasts, and microglia [62]. TREM2 is thought to act as a cell surface receptor, and although the endogenous ligand has yet to be identified, it is known that it requires the adaptor protein 12 (DAP12) for the initiation of signaling cascades [63]. Activation of the TREM2 receptor on microglia has two important function consequences: (1) stimulation of phagocytosis activity and (2) decreased microglial proinflammatory responses [64]. Collectively, TREM2 may function to help aid microglia to clear damaged or apoptotic cells and cellular debris and help resolve damage-induced inflammation.

 Insights into the important role that TREM2 plays have been deduced from individuals harboring homozygous mutations in the TREM2 gene. Such a mutation leads to The Nasu-Hakola disease, which manifests as a combination of bone cysts and dementia [65]. Affected individuals show progressive inflammatory neurodegeneration with loss of white matter and cystic bone lesions followed by death by the fifth decade of life [66].

 Further support for TREM2 being an important mediator in neuroinflammation comes from animal models of multiple sclerosis (MS). Two different studies have reported a protective role of TREM2. In one study, blockade of TREM2 function enhanced disease progression in an experimental murine model of autoimmune encephalomyelitis [67]. In the second study, intravenous application of TREM2-transduced myeloid cells limited tissue destruction and facilitated repair in a murine model of MS [68]. Taken together, these studies highlight a critical role for TREM2 during inflammatory responses in the CNS.

## 6. TREM2 in Alzheimer's Disease

 TREM2 function may affect AD pathology through phagocytosis. In a murine model of AD, Frank et al. demonstrated that TREM2 is upregulated in microglia found at the border of amyloid plaque deposits [69]. Moreover, TREM2 expression has been positively correlated with the phagocytic clearance of beta-amyloid in APP transgenic mice [70]. Given the well-documented role that microglia play in removing beta-amyloid [61], the expression of TREM2 by beta-amyloid plaque-associated microglia may be interpreted as an effort to enhance beta-amyloid clearance and to limit the proinflammatory cytokine expression in response to microglia activation by beta-amyloid itself. Besides clearing beta-amyloid, TREM2 may also function to remove debris and participate in synapse remodeling [71].

 The strongest evidence to date supporting a role for inflammation in AD comes from two recent studies demonstrating that TREM2 variants increase the risk for AD approximately 3-fold [72, 73]. These studies indicated that individuals that are heterozygous for several TREM2 mutations (the most common variant being a R47H change) were at a greater risk for AD. In addition, Jonsson and colleagues also showed that elderly carriers of the TREM2 variant who were asymptomatic for AD, nevertheless, performed worse in cognitive exams as compared to noncarriers [72]. It is noteworthy that this particular mutation is extremely rare, only being found at a frequency of 0.63 percent. Compare this to the greatest risk factor for late-onset AD, the apoE4 allele, in which it has been estimated that approximately 40% of AD subjects currently harbor at least one copy of this allele [74].

 Interestingly, it is only individuals that are heterozygous for TREM2 that are at risk for AD: homozygous, autosomal recessive mutations for TREM2 result in The Nasu-Hakola disease in some individuals characterized by bone cysts and dementia [75], while resulting in fronttemporal dementia without bone disease in others [76]. Due to the common thread of dementia in these homozygous mutations, it was these initial findings that spurred further research to search for heterozygous mutations in the TREM2 gene in AD subjects. 

**Figure 3:**
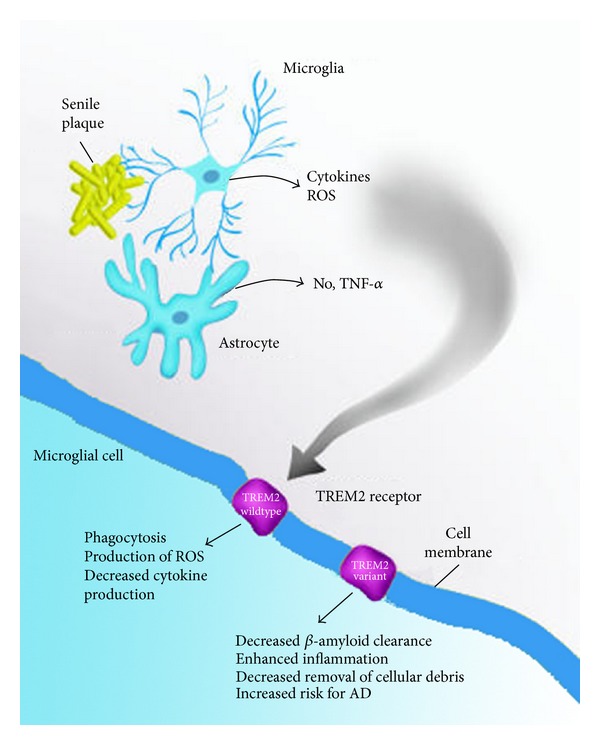
A central role for microglia in Alzheimer's disease is dependent upon a functional TREM2 receptor. Microglia represent one of the three classes of glia cells, whose primary function is to act as a major line of active immune defense in the CNS. In AD, microglia (top) and astrocytes (bottom) function in phagocytosis and in this regard help clear the brain of beta-amyloid deposits and apoptotic cells as well as any cellular debris. The important actions of microglia appear to be mediated through activation of the TREM2 receptor whose few known roles include suppressing inflammation and stimulating phagocytosis. As shown recently, variants in the TREM2 receptor have been discovered, and it has been suggested that the change in sequence leads to a loss of receptor function. It has been hypothesized that the loss of TREM2 activity has two major consequences: (1) decreased ability of microglia to remove extracellular deposits of beta-amyloid and (2) enhanced neuroinflammation. The loss of TREM2 function and altered immune responses by microglia may explain the increased risk for AD for individuals carrying the heterozygous mutations in TREM2.

Given the prophagocytic role of TREM2, loss of phagocytic activity of microglia could represent one mechanism by which the TREM2 mutations increased the risk to develop AD (Figure 3). Because TREM2 functions in microglia to also dampen microglial activation, mutations and loss of function could result in runaway inflammation as well. Based on these results, it is tempting to speculate on the potential pharmacological value that TREM2 agonists might have in AD. Future directions should answer this question as well as the possibility of finding more rare variants that have similar effects, including those in DAP12 and TREM2's intracellular signaling partner.

## 7. Concluding Remarks

 Research in the field of AD has uncovered the detailed molecular mechanisms leading to the hallmark, microscopic lesions consisting of beta-amyloid plaques and NFTs. However, how these lesions lead to neurodegeneration is still under investigation. An important consideration in this paper was the attempt to unify the various potential players that contribute to cell death in AD, including beta-amyloid, NFTs, caspases, oxidative stress, and inflammation. In this regard, it is suggested that beta-amyloid in the form of soluble beta-amyloid oligomers represents the earliest known step in the entire process, setting off a chain of events that ultimately lead to chronic inflammation and neuronal cell death. It has been difficult to assess the cause and effect relationship of inflammation in AD, but the recent discovery of the TREM2 mutations has now put inflammation back on center stage as a process that contributes to disease progression. This is highlighted by the data indicating an approximate 3-fold increase in the risk for AD in individuals harboring heterozygous variants in the TREM2 gene. Because TREM2 functions to modulate the inflammatory immune responses in microglia, mutations in this gene, in turn, could contribute to disease pathogenesis by preventing the clearance of beta-amyloid deposits and/or by enhancing inflammation. However, because the function of TREM2 is still poorly understood, it is difficult to determine how loss of TREM2 function might contribute to the disease process. Future studies examining a direct role for TREM2 in AD should help shed light on this question and provide further support for the role of chronic inflammation in this disease.

## Abbreviations

AD: Alzheimer's disease
apoE4: Apolipoprotein 4
APP: Amyloid precursor protein
NFTs: Neurofibrillary tangles
PHFs: Paired helical filaments
TREM2: Triggering receptor expressed on myeloid cells 2.
